# Biotechnological advancements enabling cannabinoid biosynthesis in engineered fungi: a mini review

**DOI:** 10.3389/ffunb.2025.1660661

**Published:** 2025-10-24

**Authors:** Madira Coutlyne Manganyi, Christ Donald Kaptchouang Tchatchouang

**Affiliations:** ^1^ Department of Biological and Environmental Sciences, Sefako Makgatho Health Sciences University, Pretoria, South Africa; ^2^ Faculty of Applied Science, Eduvos, Midrand, South Africa

**Keywords:** biotechnology, cannabinoid biosynthesis, CRISPR-Cas9, engineered fungi, synthetic biology

## Abstract

Cannabinoids, such as Δ^9^tetrahydrocannabinol (THC) and cannabidiol (CBD), are bioactive compounds with well-documented therapeutic potential, including applications in pain relief, neuroprotection, anti-inflammatory treatments, and seizure control. Traditionally sourced from *Cannabis* plants, their production remains limited by agricultural constraints, regulatory hurdles, and environmental concerns. In response, recent advances in biotechnology have enabled the microbial biosynthesis of cannabinoids, offering a scalable and sustainable alternative. Engineered fungi, in particular, have gained attention as promising production platforms due to their metabolic flexibility, ease of genetic manipulation, and capacity for synthesizing complex secondary metabolites. This mini-review explores key innovations in synthetic biology and metabolic engineering that have enabled fungal cannabinoid biosynthesis. It highlights strategies such as pathway reconstruction, enzyme optimization, host strain engineering, and the application of CRISPR-Cas9 genome editing. In addition, it examines ongoing challenges, including product toxicity, metabolic burden, and regulatory considerations. Finally, the review outlines future directions in systems biology, the production of rare cannabinoids, and bioprocess optimization. Overall, the development of engineered fungi for cannabinoid biosynthesis represents a major conceptual advance in microbial biotechnology, with far-reaching implications for the pharmaceutical, nutraceutical, and industrial sectors.

## 1 Introduction

In recent years, there has been a remarkable surge in global interest and research on *Cannabis* sativa, driven largely by the therapeutic promise and economic value of its active compounds, cannabinoids. Among these, Δ^9^tetrahydrocannabinol (THC) and cannabidiol (CBD) are the most extensively studied, known for their diverse pharmacological effects, including analgesic, anti-inflammatory, antiseizure, anxiolytic, and neuroprotective properties (Pertwee, 2006; Morales et al., 2017; Pisanti et al., 2017). This surge in popularity has paralleled the rapid growth of the global legal *Cannabis* market, which is projected to exceed USD 60 billion by 2027 (Grand View Research, 2023). Legalization trends across North America, Europe, Africa, and parts of Asia have contributed to increased consumption and normalization of cannabinoid containing products (Abuhasira et al., 2018). Cannabinoids are currently utilized across multiple industries, including pharmaceuticals, cosmetics, nutraceuticals, and functional foods (Andre et al., 2016; Hanuš et al., 2016). Medical *Cannabis* is now legal in over 50 countries, while recreational use is permitted in several jurisdictions, leading to increased research, product innovation, and commercialization (European Monitoring Centre for Drugs and Drug Addiction, 2018). Despite this progress, cannabinoid production through *Cannabis* cultivation presents several challenges—slow growth cycles, environmental variability, land and water use, and strict regulatory controls that limit scalability and standardization (Jin et al., 2017; Chakraborty et al., 2021).

To overcome the challenges of plant-based production, microbial biosynthesis of cannabinoids has emerged as a promising alternative. Fungi are gaining attention due to their fast growth, metabolic versatility, and industrial utility. Synthetic biology tools now enable the expression of cannabinoid pathways in fungi, including enzymes that produce key precursors such as for producing key precursors like olivetolic acid and geranyl pyrophosphate (Keller, 2019; Luo et al., 2019; Gülck and Møller, 2020; Vogt et al., 2021).

Moreover, breakthroughs in genome editing technologies, particularly CRISPR-Cas9, have significantly accelerated strain development and pathway optimization (Nødvig et al., 2015). Notably, recent studies have demonstrated the successful production of cannabinoids in engineered fungal strains, such as *Aspergillus niger*, laying the foundation for a cost-effective, environmentally friendly, and regulation-compliant production platform (Drysdale et al., 2022). This review provides a comprehensive overview of current advancements in fungal-based cannabinoid biosynthesis, with emphasis on engineering strategies, current challenges, and potential applications.

## 2 Synthetic biology and metabolic engineering

**Figure 1:**
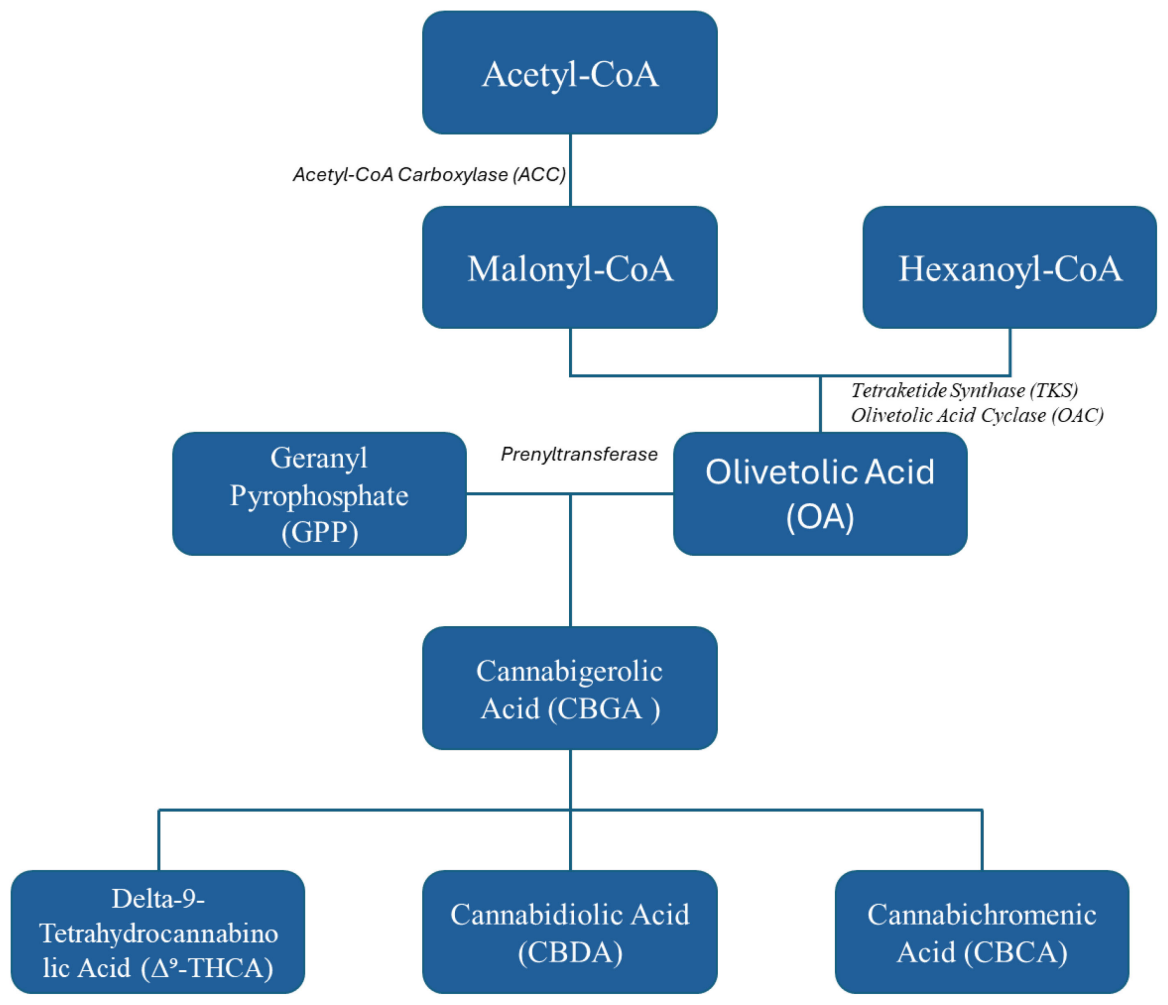
Schematic overview of the engineered biosynthetic pathway for cannabinoid production, illustrating the enzymatic conversion.

A major breakthrough in modern biotechnology has been the ability to reconstruct cannabinoid biosynthetic pathways in microbial systems using synthetic biology tools. Central to this achievement is the engineering of fungal hosts to express key enzymes from *Cannabis sativa* that are responsible for producing the primary cannabinoid precursors—olivetolic acid (OA) and geranyl pyrophosphate (GPP). These precursors combine to form cannabigerolic acid (CBGA), the parent molecule for major cannabinoids including THC, CBD, and CBC (**Figure 1**). Genes encoding enzymes such as olivetolic acid cyclase (OAC) and geranyl pyrophosphate: olivetolate geranyl transferase (GOT) have been successfully cloned and introduced into fungal platforms, including *Aspergillus niger* and *Penicillium chrysogenum*, enabling them to produce cannabinoids under controlled fermentation conditions (Russo, 2011; Qiu et al., 2022). Fungi are naturally suited for this purpose due to their established role in producting of complex secondary metabolites. Their genetic malleability, robust growth profiles, and compatibility with large-scale fermentation processes make them ideal candidates for heterologous production of cannabinoids (Pamplona et al., 2018; Santiago et al., 2019). Recent optimization strategies have included CRISPR-Cas9 genome editing, promoter refinement, and pathway balancing to improve flux toward target compounds. In some systems, these interventions have led to a 40-fold increase in CBGA yield, demonstrating the feasibility of fungal cannabinoid production at a commercially relevant scale (Ceroni et al., 2018).

Recent studies report olivetolic acid (OA) titers of up to 15.79 mg/L in engineered *Yarrowia lipolytica* (Hong et al., 2025). Engineered *Penicillium chrysogenum* strains produced CBGA at 0.67 mg/L (supernatant) and 1.51 mg/L (lysate), with olivetolic acid reaching up to 12.23 mg/L (Kosalková et al., 2023). In contrast, *Saccharomyces cerevisiae* has achieved titers of>100 mg/L 88 CBGA through pathway optimization and precursor feeding strategies. However, filamentous fungi like *P. chrysogenum* offer distinct advantages, including native polyketide synthase machinery, robust secondary metabolite secretion, and scalable filamentous growth, which make them promising long-term hosts for complex cannabinoid biosynthesis once further optimized. Despite advancements, the therapeutic equivalence of biosynthesized cannabinoids and plant-derived extracts remains a topic of debate. While microbial systems offer precision and scalability, whole-plant *Cannabis* advocates highlight the “entourage effect” a synergy among cannabinoids, terpenes, and flavonoids that is difficult to replicate with isolated compounds (Nakagawa et al., 2011; Wang et al., 2023). As such, the debate continues over whether purified cannabinoids synthesized in fungi can truly substitute the full-spectrum effects offered by plant-derived products a question that holds significant implications for drug development, regulatory approval, and clinical practice (Karasek and Stasiak, 2021).

## 3 Optimization of fungal hosts and divergent perspectives

Fungi have emerged as promising platforms for cannabinoid biosynthesis due to their metabolic versatility, industrial compatibility, and ability to produce complex secondary metabolites. Filamentous species such as *Aspergillus niger, Penicillium chrysogenum*, and *Trichoderma reesei* are of particular interest, as they are well characterized, genetically tractable, and already utilized in large-scale bioproduction (Geiselmann et al., 2022; Liu et al., 2023). These hosts possess endogenous pathways such as those for terpenoid and polyketide synthesis that align with the biochemical demands of cannabinoid production. To optimize fungal cannabinoid biosynthesis, researchers have targeted both primary and secondary metabolism. Enhancements in precursor availability, including the upregulation of the mevalonate 114 pathway (for GPP) and polyketide synthase pathways (for OA), have resulted in significant yield improvements (Keasling et al., 2022). Metabolic modeling and flux analysis are now standard tools in identifying bottlenecks and optimizing expression levels (Hudalla et al., 2024). Furthermore, synthetic promoter libraries, codon optimization, and dynamic pathway regulation systems are being deployed to fine-tune expression of cannabinoid biosynthetic genes (Stone et al., 2020). However, there is an ongoing scientific debate over the ideal production platform. While synthetic biology advocates view fungi as sustainable, consistent, and scalable, others argue that microbial systems lack the biochemical complexity of the upregulation of the mevalonate 114 pathway (for GPP) and polyketide synthase pathways (for OA), have resulted in the synergistic therapeutic interplay among cannabinoids, terpenes, and flavonoids (Almeida et al., 2023). These differing schools of thought have implications not only for technical development but also for downstream clinical acceptance and regulatory approval.

## 4 Engineering challenges and scientific disagreement

Although significant progress has been made in constructing functional cannabinoid pathways in fungi, several biological and technical challenges persist. One of the primary issues is the cytotoxicity of cannabinoids to fungal cells. These lipid-soluble compounds can integrate into membranes or disrupt cellular signaling, leading to reduced growth and metabolite accumulation (Singh and Bhatia, 2022). To mitigate this, strategies such as efflux pump expression, product sequestration in organelles (e.g., peroxisomes), and the use of tolerance-enhancing mutations are being explored (Almeida et al., 2023). Additionally, the multistep nature of cannabinoid biosynthesis requires tight coordination of gene expression. Even slight imbalances in enzyme activity can lead to precursor buildup or the formation of shunt metabolites, thereby, reducing overall efficiency. Advanced engineering techniques, such as CRISPR137 Cas9-mediated transcriptional tuning and genome-scale pathway balancing, are now being used to fine-tune these systems (Nødvig et al., 2015; Stone et al., 2020). This complexity has sparked another layer of controversy: some researchers believe that microbial systems are ill-suited for full cannabinoid biosynthesis and better suited for producing isolated or rare cannabinoids. They argue that microbial chassis may struggle to match the pharmacodynamic complexity and consumer appeal of full-spectrum *Cannabis* extracts (Russo, 2011). On the other hand, industrial stakeholders and biopharmaceutical companies see microbial systems as a clean, reproducible, and patentable solutions particularly valuable for producing minor cannabinoids like THCV and CBDV, which occur in low abundance in plants (Karasek and Stasiak, 2021; Hudalla et al., 2024).

## 5 Success stories, technological advances, and the road ahead

Despite challenges, notable successes have been achieved in engineering fungi for cannabinoid production. In 2022, a landmark study demonstrated the production of cannabigerolic acid (CBGA) in *Aspergillus niger*, achieved by expressing a full suite of biosynthetic genes and optimizing host metabolism for precursor availability (Drysdale et al., 2022). This work confirmed the feasibility of producing key cannabinoid intermediates at commercially relevant scales through the use of fungal fermentation. Cutting-edge tools, such as CRISPR-Cas9, dynamic biosensors, and synthetic gene circuits, have accelerated 155 the field, enabling researchers to modulate pathways in real-time based on metabolite levels (Ceroni et al., 2018; Geiselmann et al., 2022). These systems also enable rapid testing of cannabinoid analogues and unnatural derivatives, offering opportunities to explore next-generation therapeutic compounds that may surpass the efficacy of natural cannabinoids. Yet, success in the lab does not guarantee acceptance in the clinic or market. A growing divide exists between proponents of “natural” plant-derived cannabinoids and those favoring precision-engineered biosynthetic products. Critics caution that synthetic production might overlook the holistic pharmacology of *Cannabis*, especially in contexts where whole extract formulations are favored for their perceived broader efficacy (Russo, 2011; Stone et al., 2020). In contrast, supporters of microbial production emphasize purity, traceability, and standardization features that are particularly critical in pharmaceutical development and international regulatory environments (Hudalla et al., 2024).

**Figure 2:**
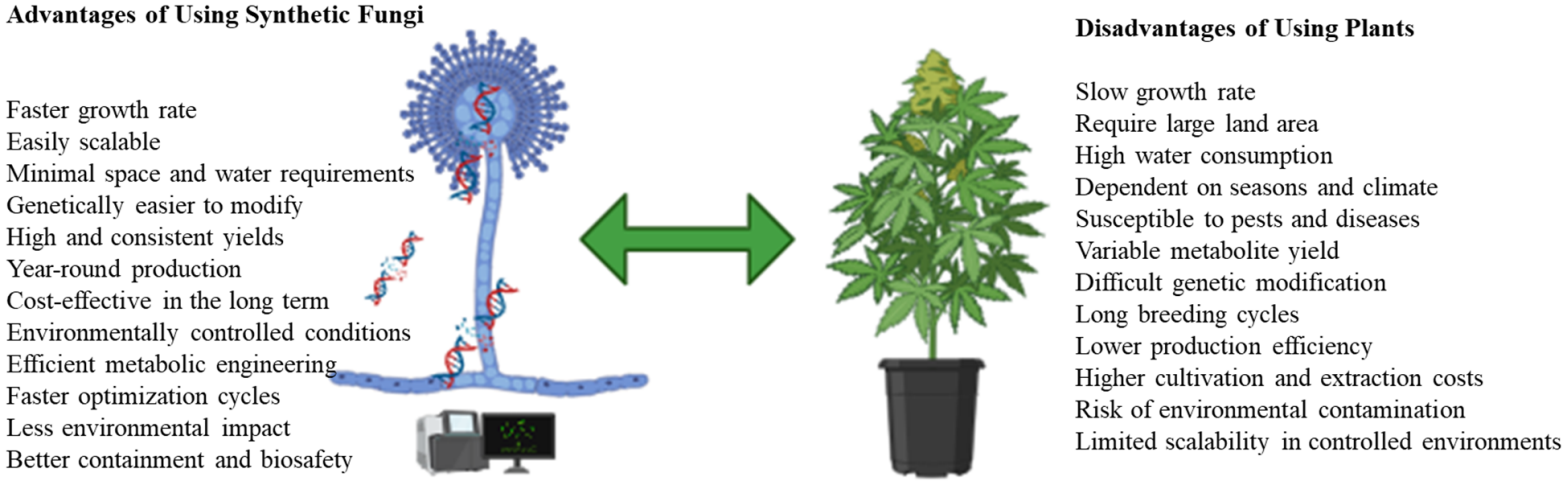
Advantages of synthetic fungi over plants in biotechnology and industrial applications (created by MC Manganyi using BioRender.com).

Synthetic fungi offer a promising platform for producing high-yield, pharmaceutical-grade cannabinoids due to their rapid growth and genetic tractability (**Figure 2**). Future strategies may combine microbial cannabinoids with plant-derived terpenes to mimic the entourage effect, thereby bridging the gap between scientific and therapeutic perspectives.

## 6 Applications and implications

The biosynthesis of cannabinoids in engineered fungi represents a transformative shift in the cannabinoid supply chain. Unlike traditional *Cannabis* cultivation, which requires vast amounts of land, energy, and water, fungal fermentation enables sustainable, high throughput production in controlled bioreactors (Liu et al., 2023). These systems drastically reduce environmental burdens and allow year-round production, independent of agricultural constraints such as climate or soil quality. Furthermore, fungi can be engineered to bypass the time-consuming maturation periods associated with *Cannabis* sativa, leading to faster turnaround times and lower production costs (Keasling et al., 2022).

Fungal platforms enable the selective expression of biosynthetic pathways for rare cannabinoids, such as cannabigerol (CBG), tetrahydrocannabivarin (THCV), and 192 cannabichromene (CBC), which are known for their diverse therapeutic potential. For instance, THCV shows promise in appetite suppression and glycaemic regulation, making it a potential candidate for obesity and type 2 diabetes treatments (Pertwee, 2006). Meanwhile, CBC exhibits strong anti-inflammatory and neuroprotective properties, which are being explored in models of neurodegenerative diseases and chronic pain (Almeida et al., 2023). The ability to engineer fungi for the precise production of such molecules accelerates drug discovery pipelines and reduces dependency on *Cannabis* biomass extraction.

Microbial cannabinoid production ensures consistency and safety but introduces new regulatory challenges. Authorities, such as the FDA and EMA, must assess the purity, bioequivalence, and long-term safety of fungal-derived cannabinoids. Ongoing debates surrounding classification and labeling may impact clinical use and global commercialization (Hudalla et al., 2024).

## 7 Future directions

Fungal engineering for cannabinoid biosynthesis is a relatively new field, but advancements in CRISPR-based genome editing, chassis development, and metabolic flux analysis are rapidly accelerating progress (Stone et al., 2020). Recent breakthroughs in utilizing of *Aspergillus oryzae* and *Trichoderma reesei* as high-yielding hosts have demonstrated enhanced cannabinoid titers through pathway optimization and precursor feeding strategies (Singh and Bhatia, 2022). Future research is expected to delve deeper into system-level modeling, enabling dynamic control over metabolic nodes to maximize cannabinoid yield while minimizing toxic byproducts. Efforts are also underway to engineer fungal strains that can utilize low-cost, renewable substrates such as agricultural waste or lignocellulose hydrolysates further enhancing sustainability (Singh and Bhatia, 2022). Integrating AI driven bioinformatics with omics data will play a pivotal role in identifying novel enzymes, regulatory elements, and gene circuits to fine-tune cannabinoid biosynthetic routes.

## 8 Conclusion

The convergence of synthetic biology, fungal biotechnology, and cannabinoid research is paving the way for a new era in pharmaceutical manufacturing. Engineered fungi not only offer a scalable and ecoconscious alternative to traditional *Cannabis* cultivation but also enable the tailored production of pharmacologically relevant cannabinoids. While regulatory frameworks and technical challenges remain, the trajectory of this field suggests that fungi may soon become central players in the biomanufacturing of cannabinoid-based therapeutics.
